# 

**Published:** 1887-04-02

**Authors:** 


### The Hospital

A WEEKLY INSTITUTIONAL JOURNAL OF

### Science, Medicine, and Philanthropy.

EDITED BY

HENRY C. BURDETT,

AUTHOR OF "HOSPITALS AND THE STATE"; "PAY HOSPITALS OF THE WORLD"; "COTTAGE HOSPITALS, GENERAL, FEVER, AND CONVALESCENT, WITH FIFTY BEDS AND UNDER: THEIR ORGANISATION, MANAGEMENT, AND WORK": ETC., ETC.

ACTING EDITOR:

GEORGE W. POTTER, M.D.,

AUTHOR OF PAPERS ON "BACTERIOLOGY"; "SCARLET-FEVER"; "RHEUMATISM"; ETC., ETC.

VOL. II. FOR 1887.

(2ND APRIL 1887 TO 24TH SEPTEMBER 1887.)

SURGEON GEN'L'S OFFICE LIBRARY 115352

LONDON:

PUBLISHED BY A. P. WATT, 2, PATERNOSTER SQUARE.

WI
NO69X

### INDEX TO VOL. II., 1887.

Aberdeen and its Hospitals, 11
——— Royal Infirmary, 43, 298, 398
Africa, First Days in, 427
After the Battle, 220
Alcohol and Milk Consumption at the Metropolitan and Provincial Hospitals, 413, 429
Alias, a Friendly, 269, 285, 303
Ambulance Association, St. John's, 40
Amusements in Hospitals, 43
Anæsthetics, Stimulus to Voluntary Effort, 62
And who is my Neighbour, 119
Animal Life, Incidents of, 82, 107, 149, 287, 322, 355, 372, 436
Anti-vaccinators, 401
Arbroath Infirmary, 332
Armour Skeleton, the, 361
Asylum, May a young Lady be detained in against her Parents' Wishes, 322
"Australian Town and Country Journal," Extraordinary Notice in, 59

Barker, Police-Sergeant, the Case of, 128
Barnsley Feast and the Hospital, 382
Bathers, Untrained, 283
Bath Hospital and Convalescent Home, Cost of, 366
Bedford, Bishop of, on Hospitals, 201
Beds, Endowed, 11
Begging Parsons, 241
Belfast, Hospital Saturday at, 415, 431
Belgians, King of, and London Hospital, 61
Belvidere, Fever Hospital, Opening of, 60
Birmingham and Midland Eye Hospital, 332
——— Children's Hospital, 299
——— Queen's Hospital, 281, 381
Blackburn Infirmary, Grand *Fête* in Aid of, 432
Blacklock Convalescent Hospital, Story of, 58
Blackpool, Victoria Hospital, 315
Bolton Hospital, Working Men's Collections on behalf of, 253
Books, Chats about New, 263, 337
——— for Hospitals, 394
——— Good and Otherwise, 52
Bootle, Borough Hospital, New Wing, 398
Bournemouth Hospital Scheme, 365
——— Street Collection for, 380
Brabazon Home of Comfort, 13
Bradford, Children's Hospital at, 281
Bridgnorth Infirmary and Mr. Horace Jefferies, 432
Bridgewater Infirmary, State Procession in Aid of, 430
Brighton Dental Hospital, 414
Bristol General Hospital, 430
——— Royal Infirmary, 25, 127, 265, 300
British Association, Annual Meeting, 391
British Lying-in Hospital, Private Wards at, 27
British Medical Association, Annual Meeting, 335
British Ophthalmic Hospital, 93
Brompton Hospital, 10, 58, 184
Building unnecessary Hospitals, a Passion for, 251
Burnley, Victoria Hospital, 314
Burslem, New Infectious Hospital for, 74
Bury St. Edmunds Hospital, 416
Butter, Adulteration of by Yorkshire Farmers, 433
Buttercups and Daisies, 209

Calcutta, Medical Registration Act for, 366
Cambridge, Duke of, at the Earlswood Asylum for Idiots, 9
——— at the Royal Hospital, Chelsea, 249
Cancer Hospital, Brompton, Patients' Entertainment at, 40
Canterbury, Archbishop of, on Hospitals, 179, 184
Cardiff Infirmary, Election of Ophthalmic Surgeon at, 77
Careless Dispensing, 235
Carmarthen Infirmary, 142
Carnarvon, Hospital Accommodation for, 218
Central Throat and Ear Hospital, 202
Charing Cross Hospital Medical School, Prize Day at, 249
Charity Organisation Society, and Convalescent Homes, 93
Charity Sermons, 399
Chats about Medical Museums, 28, 91, 125, 146, 191, 313, 352
Chats about New Books, 263, 337
Chelsea Hospital for Women, Dramatic Entertainment in Aid of, 108, 143
Chelsea, Royal Hospital, Visit of Duke of Cambridge to, 249
Children in London, Trips to the Country for, 209
Children, Invalid, 339
Children, North Eastern Hospital for, 250
Children's Descriptions of Jubilee Celebrations, 244
Children's Diarrhœa, 287
Children's Hospital at Bradford, 281
Children's Hospital, Birmingham, 299
Children's Hospital, Edinburgh Royal, 365
Children's Hospital, Great Ormond Street, 126, 143, 184
Children's Hospital, Paddington Green, 200, 216
Children's Hospitals, 309
Children, Victoria Hospital for, 26, 128, 142
Chronic Hospitals, 11
City of London Truss Society, 94
Clayton Hospital, Wakefield, 299
Clergy and Ministers of Religion, Notices to, 175
Colchester Physician, Where is the, 335
Collecting Dogs, 400, 415
Consultants, Dishonourable, 283
Consumption, a New Cure for, 187
Consumption Hospital, Ventnor, New Block for, 300
Convalescent Aid, 251
Convalescents, Amusements for, 353, 397, 426
Cork Dispensary, 299
Cornwall Royal Infirmary, 415
Costly Patients, 335
Cot, Cost of Endowment of, 298
Cottage Hospital Construction, 15
Cottage Hospital Management, Small Economies in, 23
Cottage Hospitals, 305
Counterparts, by a Student of Eastern Philosophies, 181, 195, 211, 227, 243
Cow with the Iron Tail, the, 433
Cruel Treatment of a Female Patient by her Parents, 339
Cure, a Wonderful, 301
Curiosities of Ancient Leechcraft, 98, 113

Dangerous Drugs, 277
Deaconess's Institution and Hospital at Tottenham, Prince and Princess of Wales at, 185
Devon and Exeter Hospital, 416
Devonport, Royal Albert Hospital, 10
Devonshire Hospital, Buxton, 128
Diarrhœa in Children, 287
Diary, the In- and Out-Patients Hospital, 16, 32, 49, 84, 99, 118, 134, 152, 192, 207, 223, 256, 272, 289, 306, 406, 422, 438
Disinfectants, 285
Dispensaries for Sailors, 360
Dispensers and Chemists, a Warning to Hospital, 431
Dispensing, Careless, 235
Divided House in a Cathedral City, a, 436
Doctors, a Deluge of, 351
——— and Druggists, 401
——— and Peerages and other Titles, 235
——— Death against, 67
——— Fees, 225
——— in Fiction, 252, 262, 279, 297
——— Out in the Cold, 235
——— Sceptical, 423
Doctor's Pulpit, the, a Children's Sermon, 311
——— A Holiday Sermon, 393
——— Blood-letting, 343
——— Blood-making, 359
——— Dangerous Drugs, 277
——— Fairy Rings and Ringworms, 311
——— Hay-Fever, 295
——— Hypochondriasis, 331
——— King Solomon's Physic, 409
——— Nerves, their Uses and Troubles, 253, 261
——— Rheumatism, 213
——— Scarlet Fever, 375
Donnybrook Hospital, Foundation-stone of, laid by H.R.H. Prince Albert Victor, 233
Drugs or no Drugs, 180
Dublin, Consumption Hospital, 126
——— Royal Hospital for Incurables, 282
Dundee Royal Infirmary, Concert in Aid of, 200
Dundrum Asylum, Salaries at, 316
Dynamite as a Life Preserver, 111

Earlswood Asylum for Idiots, 9
Early Rising, Cheerful Health, 46
Eastbourne, Princess Alice Memorial Hospital at, 334
Easter Eggs, 33
Edinburgh Association for Incurables, 218
Edinburgh, Royal Children's Hospital, 365
Endowed Beds, 11
Endowed Hospital, Advertisements of, 31
Epilepsy and Paralysis, Hospital for, Annual Meeting of, 109
Epilepsy, Hospital for, Regent's Park, 144
Epileptic, Home for an Incurable, 428
Essex and Colchester Hospital, 41
Ethel's Experiment, 318

Falkirk, Cottage Hospital for, 364
Farinaceous Food, 371
Ferret, a Baby Worried to Death by a, 432
Fever Cases, Disinfection in and after, 116
Fever Epidemic in Metropolis, 365, 375, 380, 400, 432
Fever Hospital, Islington, Decorative Wall Papers for, 249
Fever Hospitals and Large Towns, 310
Fever Nurses, their Dangers and Defences, 85
Fireman, a Heroic, 356
Fleetwood, Cottage Hospital for, 58
Floating Hospital on the River Tyne, 336
Flower Services and Hospitals, 258
Flowers, Ferns, and Ward Decorations, 15
Flowers for the London Poor, 227
Folkestone, Victoria Hospital, 415
Free Criticism, 43
French Hospital, an Inside View, 371
French Hospitals, Religion in, 61
Friendly Alias, a, 268, 285, 302

Glasgow Charities and Doctors, 95
——— Medical Charities, 75, 265
——— Medical Charities Committee, 415
——— Proposed New Infirmary, 300

33759

Glass Eyes, their Manufacture and Cost, 121
Gloucester General and Gloucestershire Eye Institution, 144
Good Women, 203
Gravesend Hospital, Extension of, 248
Great Northern Central Hospital, 126, 200, 232, 299, 349, 400
Grievances, a Book of, 129
Grimsby Hospital, 282
Growth of the Hospital Sunday Fund, 235
Guessing Competitions, 415
Guild of Friends of the Infirm in Mind, 329
Guy's Hospital and Southwark Teachers' Association, 400
——— Donation of £10,000 by Mr. J. S. Morgan to, 108
——— Special Appeal Fund, 332
——— Story of, 259, 275
——— Theatrical Performance in Aid of, 75

Half a Million of Money in Sight, 62
Halifax Infirmary, 60
Harley Street Establishment for Invalid Gentlewomen, 39
Harrogate, Bath and Convalescent Home at, 41
Hartlepools Hospital, the, 348
——— and its House Surgeon, 348
Hay Fever, 295
Heads, Bald, 267
Holbeck, New Wortley and District Infirmary, 383
Holidays, Wasted, 273
Home for a Lady, 305
Home for an Invalid Nurse, Wanted a, 15
Home Hospital, a New, 387
Homes for Convalescents with Wounds, 38
"Hospital," the, Where to Buy, 420
Hospital Administration.—Alcohol and Milk Consumption, 413, 429
——— Architects, 142
——— Book-keeping, 111
——— Charity, the Best Means of Preventing its Abuse, 130
——— Festivals, 382
——— Resources, Suggestions for the Improvement of, 249
——— Saturday at Belfast, 415, 431
——— Saturday at Lincoln, 216
——— Saturday Convalescent Home, 366
——— Saturday Fund, 24, 41, 71, 177, 202, 203, 399
——— Saturday in the Country, 281
——— Saturday, Suggestion that Gentlemen should Preside at the Stalls, 219
——— Supporters, 383
——— Worthies, 103, 137, 293
——— Sunday Fund, 142, 199, 235
——— Sunday Fund and Convalescent Homes for Working Men, 26
——— Sunday, Origin of, 41, 111
——— Sunday at Liverpool, 74
——— Sunday and the Queen's Jubilee, 175
——— Sunday, a Preaching Match, 193
——— Snnday at Southampton, 200
——— Sunday at the Synagogue, 214
——— Sunday Collection at Jubilee Service at Westminster Abbey, 216
——— Sunday at Newcastle, 216
——— Sunday at Worthing, 217
——— Sunday at Hull, 380
——— Sunday at Lowestoft, 380
Hospitals and London Theatres, 234
——— a Passion for Building unnecessary, 251
——— Facts about, for Preachers, etc., 197
——— Sitting on the Fence, 219
——— the Origin of, 175, 354
——— the Story of the, 123, 139, 259, 275, 345
——— Weeks, the, 145, 154, 189, 202, 204, 220, 221
Hospitals Association, 145, 280, 349, 430
——— and its Future Work, 267
——— Annual Meeting, 108
——— First Evening Meeting of Session 1887 and 1888, 414, 430
——— Last Sessional Meeting of, 109
——— Meeting at Brompton Hospital, 57
——— Reference Library, 366
Houses, Old London, and Medical Men, 254
Huddersfield Infirmary, Demonstration for Benefit of, 333
Hull and Sculcoats Dispensary, 314
Hull, Hospital Sunday at, 380
Hull Infirmary, 281
Hydrophobia, More Pasteur Failures, 401

Idiot, Nearly an, 15
Imperial Institute and Scotland, 218
Incidents of Animal Life, 82, 107, 149, 287, 322, 355, 372, 436
Incidents of the Hospitals Meetings, 221
Incurables, Royal Hospital for, West Hill, 128
India, Medical treatment of Women in, 268
———Native Medical Practice in, 387
Indifference, a Crusade against, 189, 204
Infectious Diseases and Medical Men, 317
Infectious Hospitals, 317
Infection, to avoid, 317
Insane, Reminiscences of the, 12, 96
Insanity in Childbearing, 89, 112
Italian Quack Doctor at Home, an, 3

Jefferies, Mr. Horace, 432
Journalism, the New, 373
Jubilee, the, and the Hospitals, 74, 233, 234, 248, 250, 265, 282, 300, 364
——— Celebrations, Casualties at, 264
——— Celebrations, Children's Descriptions of, 244
——— Day and the Nurses at St. Bartholomew's Hospital, 234
——— Presentation to Westminster Hospital, 248
——— Service at Westminster Abbey in Aid of the Hospitals, 250
——— and Hospital Sunday, 175
——— Tribute to Children's Hospital, Ormond Street, by Children, 184
——— Women's Offering, Disposal of Surplus of, 364, 380, 383, 405, 411

Kate Mountstephen, 368, 384, 402, 418, 434
Kensington District Nursing Association, 110
Kent and Canterbury Hospital, 316
Kilts and Physic, 51
King's College Hospital, 127
Knowledge and Thought, more Wanted, 254
Krupp Gun Wanted, a, 251

Lady Doctor, a, on Health and Disease, 132
Lager Beer, the Consumption of in New York, 432
Lambeth, Open Space for, 431
Lancaster, Infectious Hospital for, 382
Lanoline, 305
Leamington, Warneford Hospital, 399
Leeds, Ambulance Self-help at, 77
Leeds Infirmary, 299, 415
Leeds Infirmary, Convalescent Home in connection with, 10
Leicester Infirmary, 298
Lemon Juice, 283
Lenten Lilies, the Time of, 66
Let there be Light, 153
Life in Hospital, 155
Lincoln, County Hospital, 264, 299, 398, 414
Lincoln, Hospital Saturday at, 216
Liverpool, Chadwick Home Hospital at, 254
Liverpool Foundling Hospital, 75
Liverpool, Royal Infirmary, Administration of, 333
Llanfairfechan Convalescent Home, 302
Locum Tenens, 27
London Hospital, 415
London Hospital, New Nursing Home and Medical College, 144
London Hospital, the Story of the, 123, 139
London Hospital, Visit of King of the Belgians, 61
London Houses, Old, and Medical Men, 254
London, Open Spaces in, 431
London School of Medicine for Women, 94, 281
London Temperance Hospital, the, 187
Lowestoft Convalescent Home, 333
Lowestoft, Hospital Sunday at, 380
Lunatic Asylum for Middlesex, a New, 203
Lunatic Literature, 378
Lunatics, are there more now than formerly, 239
Lying-in Cases, Private Wards for, at British Lying-in Hospital, 27
Lying-in Hospital, City of London, Maternal Mortality, 300
Lying-in Hospital, Queen Charlotte's, Work of, during 1886, 94

Mackenzie, Dr. Morell, Honours for, 250
Manchester Royal Infirmary, 350
Manchester, Wilton Hospital, 10
Margate Royal Sea Bathing Infirmary, 415
Marsden, Dr. William, 103
Massage, Instruction in, 228
——— and Medical Electricity, 420
——— Moral and Physical, 21, 37, 55
Matrons and Sisters at Fifty, 133
Matron's Corner, a, 389, 405, 420
Meadows, Dr. Alfred, Death of, 58
Medical Examiners, 417
——— Men and Infectious Diseases, 317
——— Registration Act for Calcutta, 366
——— Students, 417
——— Teachers, 417
Men must do Something, 351
Merthyr, New Hospital for, 248, 265
Metropolitan Hospital, Kingsland Road, 416
Michelli, Mr. P. J., appointed Secretary to Seamen's Hospital, 232
——— Presentation to, 337
Middlesex Hospital, 201
Midwives' Institute, etc., 24, 26
Midwives, the Condition of, 321, 356
Milk, Cook your, 267
——— Dilution of, 433
Miller Hospital, Greenwich, 42
——— Jubilee Banquet at, 300
Mission Parcel Society, the, 389
Monsey, Dr. Messenger, 137
Montrose Infirmary, Convalescent Home for, 365
More Light, 276
Morgan, Mr. J. S., Donation of £10,000 to Guy's Hospital, 108
Museum at the Royal College of Surgeons, 65
Museums, Chats about Medical, 28, 91, 125, 146, 191, 313, 352
Museums, Medical, are the Public Admitted to, 228

Nairn, Hospital at, 232
National Orthopædic Hospital, 143
Nerves, their Uses and Troubles, 253
Newcastle, Children's Infirmary, the New, 110
Newcastle Infirmary, 300
Newport Infirmary, Children's Ward for, 398
New Remedies, Lanoline, 305
Newton Cottage Hospital, 298
Norfolk and Norwich Hospital, 242, 298
——— and its Chaplain, 350, 389
Norfolk, Duchess of, Death of, 59
Northampton, General Infirmary, 9
North-West London Hospital, 25
Nottingham, proposed Infections Hospital for, 431
Nottingham, proposed Throat and Ear Hospital, 334
Novel, shall we give up the, 95, 115, 135
Nurse and the Sick Room, 229, 245
Nurse in New Zealand, wanted Employment as, 15
Nurse Marion's Romance, 4, 19, 35, 53, 69, 87
Nurse, Partial Employment for a Trained, wanted, 15
Nurse, who Nurses the Sick, 267
Nurses and Nursing, "The Globe" on, 349
Nurses, Trained, Registration of, 129
Nursing Institutions, Nurses, and the Women's Jubilee Offering, 411
Nursing Institutions, Public and Private, 6, 30, 57, 104, 138
Nursing Sisters, Decoration of, by the Queen, with the Royal Red Cross, 365
Nursing the Sick, a Noble Profession, 229, 245

Out-patients' Department, the, 147, 237
Out-patients, Sponsors for, 291
Oxford, Radcliffe Hospital, Private Nursing from, 348

"Pall Mall Gazette" and New Journalism, 373
Paper, Varnished, for Hospital Walls, 65
Paralysed and Epileptic, National Hospital for, opening of three new Wards by H.R.H. the Duchess of Albany, 264
Paralysis, West-end Hospital for, 234
Parsons, Begging, 241
Pasteur, More Failures, 401
Patients, a Word to Hospital, 44
——— Admission and Discharge of, 61
——— from the Doctor's Point of View, 231

Pauper Children, who cares for the, 203
Pension Fund for Hospital Officials and Nurses, a National, 5, 27, 47, 66, 73, 88, 109, 115, 122, 141, 142, 183. 206, 214, 228 247, 254, 278, 296, 312, 328, 347, 370, 383, 383, 405, 411, 428, 430
Philanthropy as a Career, 257
Photographic Views for Birmingham Hospitals, 430
Photographs, Mounted, for Hospitals, 233
Plumbers, Registered, 367
Plymouth, Royal Albert Hospital, 300
Poetry: Queen's Jubilee, 2
——— Welcome All, 23
——— Bury me in the Churchyard at Home, 34
——— An Incantation, 39
——— Two Physicians Better than One, 45
——— One Physician Better than None, 45
——— Short and Sweet, 46
——— Song of the Iron Pill, 62
——— "The Hospital's" Nursery Rhymes, 70
——— The Medical Student's Complaint, 72
——— Saints on Earth, 102
——— The Children's Ward, 106
——— Joseph Warren, M.D., 140
——— Words for the Wise, 150, 188
——— Hospital Sunday, 205
——— What Live I for, 326
——— The Stethoscope Song, 340
——— An Army of Women, 342
——— In a Hospital Mortuary, 435
Poisoning, Wholesale, 433
Police, Convalescent Seaside Home, 300
Poor, Destruction of the, 407
Poor in Towns, the, 101
Poplar Hospital for Accidents, 185, 217
Porter, the Hospital, 95
Portsmouth, Eye and Ear Hospital and Victoria Park Flower Show, 430
Practitioners, Unlicensed, 236
Probationers, One Year, 65, 70
Provident Dispensaries, 219

Queen Charlotte's Lying-in Hospital, its Work during 1886, 94
Queen's Hospital, Birmingham, 281, 381
Quilter Prize, the, 114, 133
Queen Victoria's Bounty, 380, 383, 405

Radcliffe, John, M.D., 293
Reckless Driving, 432
Religio Medici, 327, 344, 363
Religion in French Hospitals, 61
Rentoul, Dr., on Provident Dispensaries and Hospitals, 250
Resident Medical Officers, how many should there be in Hospitals, 228
Richmond Hospital, 315
Rod of Health, the, 395
Royal College of Surgeons, Museum at, 65
Royal Hospital for Children and Women, Waterloo Bridge Road, 24, 25
Royal Red Cross, Decoration of Nursing Sisters with, by the Queen, 365
Ryan, Mr. G. Owen, appointed Secretary to Queen Charlotte's Hospital, 232
Ryan, Mr. Thomas, appointed Secretary to St. Mary's Hospital, 232

Safe from Scarlet Fever, 425
St. Bartholomew's Hospital and Jubilee Day, 2
St. John and St. Elizabeth, Hospital of, 143
St. Mary's Hospital, Presentation to Mr. Michelli, 337
——— and the Working Classes, 428
——— proposed Enlargement of, 200
——— and the Injured at the Fire at Whiteley's, 349
St. Thomas's Hospital, Alleged Inhumanity at, 307
——— Annual Old Students' Dinner, 415
——— Medical School, Prize Day at, 266
Salford, Proposal to Build an Infectious Hospital for, 317
Salisbury, Hospital Sunday at, 415
Scandal, a Hospital, 307
Scarlet Fever at Ingleston House School, 282
——— in London, 365, 375, 380, 400, 416, 432
——— Cases, Treatment of at Home, 414
——— Epidemic in London, 416
Sceptical Doctors, 423
Scepticism, Unparalleled, 335
Scrofula, Institution for Treatment of, 228
Seamen's Hospital, Greenwich, 185
Sea Water for Inland Towns, 367
Shall we give up the Novel, 95
Sheffield Hospital and Dispensary, 350
Sheffield, Hospital Saturday at, 266
Shropshire and Wales Eye, Ear, and Throat Hospital, the, 24
Sick and the Hospitals, the, 170
Sick Nurses, the "Daily Chronicle" on, 416
Sir Oracle, 283
Sir Patrick Dun's Hospital, Bazaar in Aid of, 218
Sleep and Sleeplessness, 17
Small Economies in Cottage Hospital Management, 23
Small-pox in London, 349, 382
Smith, Sydney, as a Doctor, 29
Sober Science, 391
Southend, Victoria Hospital, Bazaar and Fancy Fair, 265
——— Laying of Foundation Stone of the, 365
South Shields Friendly Societies, 282
Sponsors for Out-patients, 291
Spring, a Little Holiday in the, 1
——— some Dangers of, 90
Spurgeon, Mr., on Hospitals, 215
Story of the Hospitals, 123, 139, 259, 275, 345
Stourbridge, proposed Infectious Hospital for, 314
Stratford-on-Avon, 399
Summer Drinks, 283
Sunderland Infirmary, 218, 266, 334, 415
Sunderland Workmen's Industrial Exhibition, 415
Surgeons, Royal College of, Museum at, 65
Swansea Fever Hospital, Cost of each Inpatient, 335
——— General Hospital, 281
Swimming Baths, 367
Swindon, Infectious Diseases Hospital for, 332

Tasmania, General Hospital at Launceston, 364
Taunton and Somerset Hospital Extension Fund, 350
Tavistock Cottage Hospital, 381
Temperance Hospital, London, 400
Tewkesbury, Royal Hospital, 332
Tight Lacing, Alarming Result of, 302
Till the Doctor Comes: Every-day Ailments, 14, 45
——— About Scarlet Fever, 410
——— Trusses and Stockings, 45
Till the Doctor Comes: Infection and Disinfection, 78
——— Treatment of Emergencies, 149, 188, 239, 284, 323, 338
——— Something about Eyes, 389
Tinker or Prophet, 357
Torbay Hospital, Coroner's Inquest at, 201
——— Thank-offering to the, 280
Tweedledum or Tweedledee, 301
Two Guineas, Madam, 225
Tyne, Floating Hospital on the River, 336

University College Hospital, 348
——— Bequest by Dr. Wakley to, 8
Unlicensed Practitioners, 236
Unmarried Women, 325

Vaccination Acts at Oldham, 431
Venice, Catholic and Protestant Patients in Civil Hospital at, 416
Ventilation of Hospitals, the, 63, 79, 82, 97
Victoria Hospital for Children, 26, 128, 142
Vienna Hospital, Scandal at, 129
Visitors, Silly Complaints by, 264
Visiting Day, 377

Wakley, Dr., Bequest to University College Hospital, 8
Wales, Princess of, Visit to the Brompton Hospital, 10
Wasted Holidays, 273
Well-guessed or Well-done, 341
Wellingborough, Cottage Hospital for, 333
West Ham, proposed Hospital for, 431
West London Hospital, 348
Westminster Hospital, Jubilee Presentation to, 248
——— Out-patients' Department, 365
——— Visit of Duke and Duchess of Connaught, 334
Wigan Infirmary, 217, 233
Wimbledon Camp, Collecting Boxes at, in Aid of the London Hospitals, 216
Wirral, Children's Hospital, 314
Wolverhampton and Staffordshire General Hospital, 25
Woman's Sacrifice, a,—For Duty's Sake, 412
Women and Work, 301
Women in India, Medical Treatment of, 268
Women's Jubilee Offering, Disposal of Surplus of, 364, 380, 383, 405, 411
Women, Unmarried, 325
Wooden Hospitals, 251
Woodhall Spa, 66
Worcester Infirmary, 264
Words of Consolation: Death, 2, 18, 34, 53, 68
——— The Work of the Holy Ghost, 86, 102, 120, 136, 178, 194, 210, 226, 242, 258, 274, 292
——— The Sufferings of Christ, 308, 326, 342, 358, 374, 392, 408, 424
Working Classes, Co-operation among the, 187
Working Men and their Manners, 383
Working Women, their Present and their Future, 302

Year's Work in the Hospitals and Medical Charities of London, 171
Yorkshire Farmers, Honest, 433

Zanzibar, Hospital for, 315

### The Hospital.

**An Institution, Family, and Parochial Journal of Hospitals, Asylums, and all Agencies for the Care of the Sick, Criticism and News.**

SATURDAY, APRIL 2ND, 1887.

### The Literary Prize.

A PRIZE of **Two Guineas** will be given every month for the best story or incident based upon hospital, or asylum, or student life, or any incident connected with the professions of medicine or nursing.

### Hospital Housekeeping.

FORMS and full particulars of the Quilter Prizes under this head (see page 355 for 19th Feb.) may be had on application to the Editor of THE HOSPITAL, Norfolk House, W.C. All competitive essays to be sent in by 30th April, 1887. Prizes, £10 10s. and £5 5s.

### Flowers, Ferns, and Ward Decorations.

### Questions and Answers.

All questions should be addressed to Mr. Howard J. Collins, Secretary, the Hospitals Association, Norfolk House, Norfolk Street, Strand, W.C.

### WANTED, EMPLOYMENT AS A NURSE IN NEW ZEALAND.

Miss Catton writes:—"Can you give me any information as to how I could best hear of an engagement as trained nurse in a private nursing institution, or as assistant nurse in a hospital, with a view to becoming charge nurse, anywhere in New Zealand? Do you know if nurses are much in demand there? I shall be much obliged if you can give me any information."

*** There is no way by which a nurse can make sure of being employed in one of the hospitals in New Zealand. All such appointments are made in the Colony, and it is not known that nurses are ever specially sent for from England. Selections and appointments are certainly not made over here.

### WANTED, A HOME FOR AN INVALID NURSE

"An Anxious Matron" writes:—"Could any of the readers of The Hospital tell me what to do in the following case? A nurse has been under my care for the last two years, and for some time has been suffering from an ulcerated stomach and weak heart. She is now totally unfit for hospital work, and her medical attendant says she must have several months' entire rest. For the last six weeks she has been sent to a convalescent home in New Brighton at the expense of the hospital. I am anxious to find a place where she could be received, or should be glad to know if anyone could suggest a home or hospital, where she could be taken in, as the poor girl is without home, or friends to look after her."

### WANTED, PARTIAL EMPLOYMENT FOR A TRAINED NURSE.

A "Constant Reader" writes from Newcastle:—"Is there any dispensary or other medical institution where a nurse could be employed for half or a portion of a day?"

### COTTAGE HOSPITAL CONSTRUCTION.

Could any of your readers kindly inform me of some plan or place to gain information for building a Cottage Hospital? The inhabitants (four thousand) and other friends of Huntly have gathered about £1,200 for a Cottage Hospital, and we wish to expend it to the best advantage. Any information to that effect will be most thankfully received by

Hudson Teape, B.A., Clk.

Parsonage, Huntly, N.B.

*** The second edition of *Cottage Hospitals*, published by J. and A. Churchill, New Burlington Street, London, W., gives full information on the subject, and contains plans also.

### NEARLY AN IDIOT!

Mrs. Pryor, 17, Avenue Road, Regent's Park, wishes to know how to get a girl, aged fourteen, nearly an idiot, if not quite, into an Idiot Asylum. Parents are very poor working people, and unable to pay anything towards her maintenance. Which would be the proper asylum for a girl living at Bethnal Green?

*** Application should be made to Relieving Officer of the district, who will supply the necessary forms for signature by the parent, two medical men, and a magistrate. There is an asylum at Bethnal Green (Dr. John Millar, Bethnal House, Bethnal Green).

### The In- and Out-Patients' Hospital Diary.

This Diary is so arranged as to make some portion of it appear every week, and the whole in each monthly part. It has been very difficult to compile, and corrections will at all times be gratefully received. It has been suggested that similar information about the larger Provincial and Scotch Hospitals would be useful to many readers. We should be glad to hear if this is felt to be the case.

### I.—GENERAL HOSPITALS.

| Hospitals and Terms of Admission. | Physicians, Surgeons, and Medical Officers, with Days and Hours of Attendance. |
|---|---|
| Charing Cross Hospital, West Strand, W.C. Sec., Mr. A. E. Reade<br>Urgent cases are immediately admitted as in-patients. Subscriber's letter expected in other cases<br>Out-patients admitted without subscriber's letter first time; for continued attendance they must be obtained from the Secretary | *In-Physicians*, Drs. Pollock, M. Th.; Green, W. S.; Bruce, Tu. F. Hour, 1.30<br>*In-Surgeons*, Messrs. Barwell, M. Th.; Bellamy, Tu. F.; Bloxam, W. S. Hour, 2<br>*Out-Physicians*, Drs. Wilcocks, M.Th.; Abercrombie, Tu. F.; Lubbock, W. S.; Murray, W. S. Hour, 1.30<br>*Out-Surgeons*, Messrs. Cantlie, M.Th.; J. H. Morgan, Tu. F.; Stanley Boyd, W. S. Hour, 1.30 |
| "Dreadnought" Hospital (Seamen's Hospital Society), Greenwich, S.E. Sec., Mr. W. T. Evans<br>Entirely free to seamen and to all accident cases | *In- and Out-Physicians*, Drs. Curnow, Tu. Th. S.; H. T. Griffiths, M.W.F. Daily, between 9 and 3<br>*In- and Out-Surgeons*, Messrs. W. J. Smith, G. R. Turner. Daily, between 9 and 3 |
| French Hospital, 10, Leicester Place, Leicester Sq., W.C. Sec., Mr. E. Rimmel<br>Free to urgent cases and to all foreigners speaking French | *In-Physician*, Dr. Vintras, Tu. S. 10<br>*In-Surgeons*, Sir W. MacCormac, Tu. 9; Mr. J. Késer, W. F. 10<br>*Out-Physician*, Dr. Vintras, Tu. S. 10<br>*Out-Surgeons*, Messrs. H. de Méric, M. Th. 10; J. Késer, W. F. 10 |
| German Hospital, 113, Dalston Lane, E. Sec., Mr. C. Feldmann<br>Free to natives of Germany, and others speaking German, and to all accidents and emergencies. Others by Governors' letters<br>——— Eastern Branch, 49, Mansell Street, Aldgate, E.<br>——— Western Branch, 259, Oxford Street, W. | *In-Physicians*, Drs. Weber, M. T. 2; Port, Tu. F. 9 a.m.<br>*In-Surgeons*, Drs. Lichtenberg and Burger, W. S. 2<br>*Out-Physicians*, Drs. Tuchmann, Tu. W. 2; Ludwig, M. Th. 2; Keller, Tu. W. F. 2; Philippi, M. Th. F. 2 (*Men*, Tu. Th.; *Women*, M. W. F.)<br>*Out-Physician*, Dr. C. Harrer, Tu. F. 8 to 10 a.m.<br>*Out-Physician*, Dr. M. Castaneda, M. T. 8 to 10 a.m. |
| Great Northern Central Hospital, Caledonian Road, N. Sec., Mr. W. T. Grant<br>Free to all, without letters of recommendation | *In-Physicians*, Drs. Cholmeley, Cook, and Burnett, M. Tu. W. Th. F. 2<br>*In-Surgeons*, Messrs. W. Adams, W. Spencer Watson, and J. Macready, M. Tu. W. Th. F. 2<br>*Out-Physicians*, Drs. Beale, M. Th. 2; Beevor, Tu. 2, S. 10<br>*Out-Surgeons*, Messrs. Lockwood, M. Th. 2; Makins, Tu. F. 2 |
| Guy's Hospital, St. Thomas's Street, S.E. Supt., Dr. C. J. Steele<br>Patients are admitted, if beds are available, without letters of recommendation, on application at the Hospital. Out-patients are seen free without letters of recommendation | *In-Physicians*, Drs. Pavy, Tu. F.; Pye-Smith, M. Th. S.; Taylor, M. Tu. Th. F. Hour, 1.30<br>*In-Surgeons*, Messrs. Bryant, M. Th. Durham, M. Th. F.; Howse, W. S.; D. Colley, M. Th. Hour, 1.30<br>*Out-Physicians*, Drs. Carrington, W.; Hale White, M.; Goodhart, M. Tu. Th. F. Hour, 12.30<br>*Out-Surgeons*, Messrs. Lucas, Th.; Golding Bird, S.; Jacobson, W.; Symonds, M. Hour, 12.30 |
| King's College Hospital, Portugal Street, W.C. Sec., Mr. T. Mosse Macdonald<br>Accidents and urgent cases free at all times. Other cases by Governors' letters, but where not obtainable, application may be made to the Secretary | *In-Physicians*, Drs. Beale, Duffin, Yeo, Tu. W. F. 1.30; Tu. W. F. S. 2<br>*In-Surgeons*, Mr. Wood, Sir J. Lister, Messrs. H. Smith, Cheyne, W. Rose, Barrow. Daily, 1.30<br>*Out-Physicians*, Drs. Ferrier, J. Curnow, N. I. C. Tirard; *Men*, Tu.Th.; S. 1; *Women*, M. W. F. 1<br>*Out-Surgeons*, Messrs. W. Rose, W. Cheyne; *Men*, Tu. Th. S. 1; *Women*, M. W. F. 1 |
| London Hospital, Whitechapel Road, E. Sec., Mr. A. Haggard<br>Accidents and urgent cases free; out-patients for dental and maternity departments need no recommendation; other cases by subscriber's order. Out-patients are required to attend only once, unless otherwise directed by the Physician or Surgeon | *In-Physicians*, Drs. Mackenzie, Tu. F.; Down, Tu. F.; H. Jackson, M. Th.; Sutton, M. Th.; Fenwick, Tu. F. 1.30<br>*In-Surgeons*, Messrs. Rivington, Tu. F.; Couper, Waren Tay, M. Th.; McCarthy, W.S.; Treves, Tu.F. Hour, 1.30<br>*Out-Physicians*, Drs. Sansom, Th.; M. Ralfe, M. Th.; Turner, Tu. F.; Warner, Tu. F.; Gilbart Smith, W. S. J. Anderson, W. S. Hour, 1.30<br>*Out-Surgeons*, Messrs. Mansell-Moullin, M. Th.; Eve, M. W.; Reeves, Tu. S.; H. Fenwick, Tu.F. Hour, 1.30 |
| London Temperance Hospital, Hampstead Road, N.W. For treatment without alcohol. By letter or small payment | *In- and Out-Physicians*, Drs. J. Edmunds, M.; J. J. Ridge, Tu. F. Hour, 1.30<br>*In- and Out-Surgeons*, Mr. A. P. Gould Th. 2.30 |
| Metropolitan Free Hospital, Kingsland Road, N. Sec., Mr. G. Croxton<br>Admission free to the sick poor, without any letter of recommendation. Urgent cases at all times | *In- and Out-Physicians*, Drs. Drysdale, Tu. F. 12; J. G. Dudley, W. S. 12; Fotherby, M. Th. 12; H. H. Tooth, Tu. F. 9; A. Haig, W. S. 9; A. Davies, M. Th. 9<br>*In- and Out-Surgeons*, Messrs. E. J. Chance, W. S. 9; Goodsall, Tu. F. 9; Walsham, M. Th. 9; A. A. Bowlby, F. 9; S. Paget, Th. 9; C. J. Thompson, daily, 9 |
| Middlesex Hospital, Berners Street, W. Sec. and Supt., Mr. A. O. D. Bartholeyns<br>Urgent cases admitted at all times; other cases by ticket. All medical cases must, in the first instance, be seen by the Resident Medical Officer, who attends at the hospital daily, at 11 a.m. | *In-Physicians*, Drs. Cayley, M. Th.; Coupland, Tu. F.; D. Powell, M.Th.; Finlay, Tu. F. Hour, 1.30<br>*In-Surgeons*, Messrs. Hulke, M. Th.; Lawson, M. Th.; Morris, Tu. F. Hour, 1.30<br>*Out-Physicians*, Drs. Fowler, Tu. F. 3.30; Biss, M. Th. 1.30; Pringle, W. S. 1.30<br>*Out-Surgeons*, Messrs. Andrew Clark, M. 1.15 (*Men*); Th. 1.15 (*Women*); Pearce Gould, F. 1.30 (*Men*); Tu. 1.30 (*Women*); J. B. Sutton, W. S. 9 |
| Miller Hospital and Kent Dispensary, Greenwich, S.E. Hon. Sec. Mr. W. Bristow. | *Physicians*, Drs. T. Creed, C. H. Hartt, G. Willes<br>*Surgeons*, Messrs. T. Moore, J. Poland, E. Clarke |
| North-West London Hospital, 18, Kentish Town Road, N.W. Sec., Mr. A. Craske<br>By subscribers' tickets or payment, but accidents and urgent cases free at all times | *In- and Out-Physicians*, Drs. W. C. Hood, J. Shaw, Edwarde H. Campbell. Daily, 1.30<br>*In- and Out-Surgeons*, Messrs. F. Durham, Collier, Jennings, J. Black. Daily, 1.30 |
| Ospedale Italiano, 41, Queen Square, Bloomsbury. Sec., Sig. L. Bonacina<br>Free to all, but Italians have preference | *In- and Out-Physicians*, Drs. M. Castaneda, Tu. F. 10 to 11; B. Sassella, M.Th. 10 to 11; Mr. J.W.B. Steggall, W.S. 4 to 5 |
| Poplar Hospital, 303, East India Dock Road, E. Sec., Lt.-Col. Feneran<br>Free to accidents and emergencies | *In-Surgeons*, Messrs. F. M. Corner, M. Brownfield, and Resident Surgeons<br>*Out-Surgeons*, Dr. J. D. Grant, W. S. Mr. T. E. Bowkett, Tu. F. 12 to 2 |
| Royal Free Hospital, Gray's Inn Road, W.C. Sec., Mr. J. S. Blyth<br>Admission free to the sick poor, without any letter of recommendation | *In- and Out-Physicians*, Drs. Cockle, M. Th.; Sainsbury, Tu. F.; West, W. S. Hour, 1<br>*In- and Out-Surgeons*, Messrs. Rose, M. Th.; W. Gant, Tu. F.; Barrow, W. S. Hour, 1 |
| St. Bartholomew's Hospital, Smithfield. Clerk, Mr. W. Cross<br>Admission free, without letter or ticket. Accidents and other urgent cases admitted at any time; ordinary cases of disease daily, between 9 and 10. Patients are seen for Electrical treatment on M. Tu. Th. and F. 1.30 | *In-Physicians*, Drs. Andrew, Church, Gee, Sir D. Duckworth. Daily, 1.30<br>*In-Surgeons*, Messrs. Savory, T. Smith, Willett, Langton, M. Baker. Daily, 1.30<br>*Out-Physicians*, Drs. Hensley, M. Th. 11; Brunton, Tu. F. 11; Legg, W. S. 11; N. Moore, Tu. W. Th. S. 9<br>*Out-Surgeons*, Messrs. Marsh, Tu. F.; 12; Butlin, M. Th. 12; Walsham, W. S. 12; Cripps and Bruce Clarke, daily, 9<br>*Casualty Physicians*, Drs. Davies, Tu. W. F. S.; O. Browne, M. Tu. Th. F.; Nias, M. W. Th. S. Hour, 9 |
| St. George's Hospital, Hyde Park Corner, S.W. Sec., Mr. C. L. Todd<br>Free to accidents and emergencies. Other in-cases by Governor's or subscriber's letter, but, in necessitous cases, this recommendation is not strictly insisted on. Letters are not required for out-patients. Vaccination on Th. at 11 | *In-Physicians*, Drs. Wadham, M. F.; Dickinson, M. Tu. Th. F.; Whipham, Tu.W. S.; Cavafy, Tu. S. Hour, 1<br>*In-Surgeons*, Messrs. Holmes, M. Th. F. 1, W. 1.30; Rouse, M. Th. F. 1, W. 1.30; Haward, Tu. Th. S. 1, W. 1.30<br>*Out-Physicians*, Drs. Ewart, M. F.; Owen, Tu. S. Hours, 10.30 to 12<br>*Out-Surgeons*, Messrs. Bennett, M. F.; Dent, Tu. S. Hours, 10.30 to 12 *Men*, F. S.; *Women*, M. Tu. |

### THE CHILDREN'S CORNER.

**Third Quarterly Prize Competition.**

Begins 2nd Apr., 1887 ; ends 25th June, 1887.

PRIZES.

FOR MOST CORRECT ANSWERS TO PUZZLES.

| | | | |
|---|---|---|---|
| 1st .. | .. Thirty Shillings | 3rd .. | .. Ten Shillings |
| 2nd .. | .. Twenty „ | 4th .. | .. Five „ |

FOR NEATEST ANSWERS TO PUZZLES.
Five Shillings.

FOR BEST LITTLE LETTERS.
Ten Shillings.

**Puzzles—First Week.**

```
      *
    . * .
  . . * . .
* * * * * * *
  . . * . .
    . * .
      *
```

No. 1. DOUBLE MESOSTICH. The beginning of pride ; a li tle hovel ; giddy or the *sobriquet* of a famous statesman ; what you are trying to find out ; something we associate with valley ; a numeral ; the end of a kiss.

No. 2. ENIGMA Find in the following the name of a famous general :—

My *first* is deep ; my *second* skims the wave ;
My *third* is heavy ; and my whole was brave.

No. 3. FLOWER PUZZLE. Half a carman and a whole country form the name of a beautiful flower. What is it ?

No. 4. CONUNDRUM. What is that which went to India and stopped there and came back again because it never went there ?

No. 5. MISSING VOWEL PUZZLE. A s×××l t×××××e m×××s a w××e h××d.

**Letters—First Week.**

The Letter-subject for next week will be "Kindness to Animals."

**Results of Twelfth Competition—Puzzles.**

No. 62. CONUNDRUM. A thorn in the foot.

No. 63. TRANSPOSITION PUZZLE. Juliet; Rosalind; Miranda.

No. 64. REBUS. The bees flew after him and stung him again and again.

No. 65. BURIED NAMES. THE HOSPITAL.

No. 66. ENIGMA. This can be turned into sense by punctuating thus: "I saw a peacock ; with a fiery tail, I saw a comet ;" etc.

*All right*—Mikado, Tim, Skye, Scrooge, Isobel, P.S., A. C. K., Joe, Dot ; *Four right*—Florrie, Sutton, Paul Methven, Agatha, Alsie, Peeler ; *Three right*—Ellie, Little Mary, Bismarck, Ariadne.

**Letters.**

We start to-day on another competition for all the prizes in the Children's Corner. I hope that my little friends, whether they have won prizes or not in the competition which has just closed, will again compete, and that there will also be many fresh competitors. I have to thank some who have so very kindly introduced THE HOSPITAL to the notice of their friends, and I have no doubt that others will help me in like manner. The Prize Results will appear next Saturday.

The letters this week are really all excellent. Scrooge describes Manchester with its fine buildings, while Isobel writes a most interesting letter about Lincoln and its beautiful Cathedral. Mikado touches on some of the historical reminiscences of Leicester, and Tim, Halford, and Ellie, deal with the suburbs of the "big city" in which they reside Here is a letter from Peeler about a town whose name alone points to the days when the Romans occupied our island :—

DEAR MR. EDITOR,—Chester is a very interesting old town As one drives through the town, one cannot help admiring the very quaint black and white houses which make this town so picturesque. There is an old Roman wall which encircles the whole town, and is one of its chief curiosities. There is a museum, now open, where the public can see, amongst other interesting objects, many old Roman remains found at and about Chester. The Cathedral is well worth seeing, with its window at the West entrance, illustrating "the Resurrection of the Body of Our Lord." Many interesting old buildings can be seen at this fine old town of Chester, which would take too long to describe here.

Eaton, Chester. Yours truly, PEELER, aged 17.

Florrie thus writes me about Dulwich :—

DEAR MR. EDITOR,—I think the subject that you have chosen for us to write about this week is an extremely nice one. There are such pleasant walks in the neighbourhood in which I live. One can walk to the Crystal Palace in less than an hour. Leading to it is a beautiful avenue, thickly wooded with trees, which seem to meet together, forming quite an archway. The scenery of Dulwich has been partially spoiled by the land being so much built upon. During the last two years, a large infirmary has been built in Dulwich Grove, and not far from it, is the Dulwich Picture Gallery which is so well known.—I am, yours very sincerely, FLORRIE, aged 14.

20, St. John's Villas, East Dulwich.

Old castles are always interesting, but that of Rochester is especially so. Skye writes :—

DEAR MR. EDITOR,—I live almost on the boundary line between Rochester and Chatham. I like Rochester much better than Chatham, because of its fine old castle called a ruin, but it does not look a bit like a ruin till you are quite close to it. It is nearly covered with thick ivy, and inhabited by flocks of pigeons who follow the visitors round about the castle gardens for crumbs. Some people buy Indian corn, and take it for them on purpose. The Medway sweeps round the foot of the castle with a lovely bend with a fine bridge, under which pleasure boats and canoes are always to be seen passing. Rochester Castle was built by William I, and is consequently very old, but still looks fine and strong. I only wish you would come down and see it in summer, and let me have the pleasure of showing you round it, and its well-kept gardens (kept by a very crusty old man). It belongs now to the corporation of Rochester. Chatham is full of soldiers and sailors, and dirty people. I have no more to say about it.

I remain, yours faithfully, SKYE, aged 14.

4, Milton Terrace, Chatham.

One mark each : Peeler, Isobel, Scrooge, Skye, Tim, Florrie, Ellie, Mikado, Halford.

**Rules.**

Only one side of the paper should be written upon.

The address and full Christian name and surname, and the age of the competitor, must be stated in all replies. A *nom de plume* may be added for the purposes of publication.

The first prize may not be taken by the same competitor more than once in any one year. In the event of the winner of the first prize again scoring highest, the second prize will be awarded, the next competitor receiving the first.

Answers must be addressed to the "PUZZLE EDITOR," THE HOSPITAL, Norfolk House, Norfolk Street, Strand, W.C., not later than Thursday next. The "Children's Corner" Coupon (see advertisement pages) must be enclosed.

### AMUSEMENTS WITH PROFIT.

### PRIZE COMPETITIONS.

**No. 21. Double Acrostic.**

A *thane of Fife*, in Shakespeare's plays you'll find ;
A mystic *sword* 'tis said King Arthur wore ;
A witty *clown ;* philosopher-buffoon ;
A *stone*, the key to hieroglyphic lore ;
An Irish town, the *seat of chiefs* it means ;
A *swineherd* born ; a *conqueror* he died ;
O'er Egypt, with fair Isis, ruled this king ;
Aladdin's luck this *vessel* did decide ;
A giant *peak* among the Andes stands ;
In vain attempt to gain Guienne he fell ;
A powerful fleet we scatter'd far and wide ;
A Swiss canton for watches known full well.
(Initials and finals give the name of a London Hospital).

**No. 22. Medicine, its Use and Abuse.**

Credit will be given for the best paragraphs on the above subject. No answer may exceed 150 words.

**Answers.**

No. 17. DOUBLE ACROSTIC.

| | | |
|---|---|---|
| H | ippocrate | S |
| O | rsin | I |
| S | ali | C |
| P | ickwic | K |
| I | tali | C |
| T | riglyp | H |
| A | lbat | I |
| L | and o' the Lea | L |
| S | aint Davi | D |
| F | aki | R |
| O | liv | E |
| R | ave | N |

Correct answers have been received from Condorcet, Ellice, Gip, Mater, Paddy, Ostrich, May, A. M. E., Gretta.

No. 18. HOSPITAL.

Some of our correspondents have traced the history of this word with considerable ability and industry, and we hope to be able, later on, to publish some of the replies sent in.

Best answers have been received from Paddy, K. M., Ellice, Mater, Condorcet, Ostrich, Gip, Peeler, Perseverance, Gretta, A. M. E., Fred. C.

Answers must be addressed to THE PRIZE EDITOR, AMUSEMENTS WITH PROFIT, THE HOSPITAL, Norfolk House, Norfolk Street, Strand, W.C., not later than the *first* post on Saturday next. The full name and address must in all cases be given, but a *nom de plume* may be added for publication. Only one side of the paper should be written on. The "Amusements with Profit" Coupon (see advertisement pages) must be enclosed with replies.

